# Pulmonary function and high-resolution computed tomography examinations among offshore drill floor workers

**DOI:** 10.1007/s00420-017-1281-4

**Published:** 2017-12-01

**Authors:** Niels E. Kirkhus, Øivind Skare, Bente Ulvestad, Trond Mogens Aaløkken, Anne Günther, Raymond Olsen, Yngvar Thomassen, May Brit Lund, Dag G. Ellingsen

**Affiliations:** 1 National Institute of Occupational Health, P.O. Box 8149 Dep, 0033 Oslo, Norway; 2 Department of Radiology and Nuclear Medicine, Oslo University Hospital, Oslo, Norway; 3 Department of Respiratory Medicine, Oslo University Hospital, Oslo, Norway; 4 Institute of Clinical Medicine, University of Oslo, Oslo, Norway; 5 Present Address: Municipality of Horten, P.O. Box 10, 3191 Horten, Norway

**Keywords:** Oil mist, Diurnal variation, Follow-up

## Abstract

**Purpose:**

The aim of this study was to assess short-term changes in pulmonary function in drill floor workers currently exposed to airborne contaminants generated as a result of drilling offshore. We also aimed to study the prevalence of pulmonary fibrosis using high-resolution computed tomography (HRCT) scans of another group of previously exposed drill floor workers.

**Methods:**

Pulmonary function was measured before and after a 14-day work period in a follow-up study of 65 drill floor workers and 65 referents. Additionally, 57 other drill floor workers exposed to drilling fluids during the 1980s were examined with HRCT of the lungs in a cross-sectional study.

**Results:**

The drill floor workers had a statistically significant decline in forced expiratory volume in 1 s (FEV_1_) across the 14-day work period after adjustment for diurnal variations in pulmonary function (mean 90 mL, range 30–140 mL), while the small decline among the referents (mean 20 mL, range − 30 to 70 mL) was not of statistical significance. Larger declines in FEV_1_ among drill workers were associated with the fewer number of days of active drilling. There were no signs of pulmonary fibrosis related to oil mist exposure among the other previously exposed drill floor workers.

**Conclusion:**

After 14 days offshore, a statistically significant decline in FEV_1_ was observed in the drill floor workers, which may not be related to oil mist exposure. No pulmonary fibrosis related to oil mist exposure was observed.

## Introduction

The drill floor is the heart of any oil and gas drilling rig. This is the place where drillpipes are added to the drill string, the drilling engine is located and the drill fluid (mud) is pumped into the drill hole. The mud pumps, mud tanks (mud pits) and mud handling area are located in the proximity of the drill floor. Drilling is carried out by drill crews, where the drill floor workers are engaged in manual work at the drill floor and in the mud handling area. Drill crews working in the Norwegian offshore oil installations are transported to and from the rigs by helicopters.

Drill floor workers are exposed to mud, and historically such exposure has been surveilled by measurements of oil mist and oil vapor (Davidson et al. 1988; Eide 1990; James et al. 2000; Gardner 2003; Bråtveit et al. 2009; Galea et al. 2010). Mud is essential for drilling operations, e.g., by stabilizing the drill hole or by removal of drill cuttings. Mud is either water- or oil-based, and both types have complex chemical compositions (OGP/IPIECA, Drilling Fluid Task Force 2009). Mud circulates between the drill hole and drilling rig. During drilling, the returning mud is separated from the cuttings in the mud handling area of the rig with the use of shale shakers (Kirkhus et al. 2015).

Exposure to oil mist in the Norwegian offshore sector has decreased since the 1980s, from around 4.3 mg/m^3^ (arithmetic mean) in the time period from 1989 to 1997 to 0.54 mg/m^3^ during 1998–2004 (Steinsvåg et al. 2006). A recent study showed median oil mist concentrations of 0.18 mg/m^3^ during 2010 and 2011 (Kirkhus et al. 2015). Also, non-volatile mud components (NVM) are present in the working atmosphere of the drill floor workers (Hansen et al. 1991; Kirkhus et al. 2015). This exposure has not been regularly assessed, although oil-based and water-based drilling fluids are alkaline solutions, and mud aerosols may have alkaline properties (Hansen et al. 1991; OGP/IPIECA, Drilling Fluid Task Force 2009). Little is known about the potential lung disorders caused by exposure to oil mist, other mud components or other airborne chemical exposures such as strong cleaning fluids among drill floor workers. There are also only few studies of pulmonary health available from other industries where oil mist exposure occurs. Increased prevalence of respiratory symptoms, reduced forced vital capacity (FVC) and increased prevalence of pulmonary fibrosis have been reported among car manufacturing industry workers exposed to oil mist generated from oil-based metal working fluids and cable manufacturing industry workers (Skyberg et al. 1986, 1992; Greaves et al. 1997; Eisen et al. 2001). Animal studies have shown accumulation of macrophages with vacuolization in the alveoli and pulmonary fibrosis after oil mist exposure (Skyberg et al. 1990; Dalbey 2001).

The formation of oil particles during oil drilling is a combination of mechanical and thermal generation (Kirkhus et al. 2015). Mechanically generated aerosols contain all the chemical components present in the drilling mud. This includes, e.g., brine, emulsifiers, lime and sometimes wetting agents (OGP/IPIECA, Drilling Fluid Task Force 2009). In contrast, thermally generated particles contain only evaporated and recondensated components.

This study is part of a larger investigation of drill floor workers’ exposure and pulmonary health (Kirkhus et al. 2015). The aim of this study was to assess whether current exposure to airborne contaminants during oil drilling with oil-based mud is associated with short-term changes in pulmonary function in drill floor workers (Part 1). We further aimed to assess if previous exposure have induced pulmonary fibrosis, using high-resolution computed tomography (HRCT) in another group of drill floor workers with employment including the time period between 1980 and 1990 (Part 2).

## Materials and methods

### Study design and participants

Part 1 of the study was a 14-day follow-up of currently exposed male drill floor workers and non-exposed referents employed at oil drilling rigs in the Norwegian North Sea offshore sector. Due to the considerable logistical challenges of performing such a study, only employees working on oil rigs served by helicopter transport from Sola airport (Stavanger, Norway) were considered for participation. Seven companies at altogether six moveable and four stationary oil rigs agreed to participate in the study.

The participants were examined at the heliport of Sola airport before transport by helicopter to the rigs. Drill floor workers and referents were successively recruited at the outbound flight until the target number of 65 currently exposed workers and 65 referents were achieved. They were scheduled to be re-examined when they returned after 14 days’ work offshore. Only two participants could be examined for each flight, due to limited time between helicopter transport and connecting transport to and from the participants’ home. Exposure data were collected during the offshore stay of the drill floor workers.

The timing of the health examinations depended on the time of helicopter transport to and from the rigs. For logistic reasons, the helicopter transports the new crews offshore before the old crew is transported onshore. It was, therefore, anticipated that it could be difficult to examine the participants at the same time of the day, although this was tried to avoid an impact of diurnal variation on the pulmonary function measures. This was, however, not possible, and the pulmonary function estimates adjusted for diurnal variation are presented.

Drill floor workers manage the manual procedures of drill pipe assembling during drilling and disassembling during retraction of the drill string, at the drill floor. Other work tasks at the drill floor may be, e.g., handling the drill engine or cleaning up spills with pressure washers. In addition, drill floor workers run the shale shakers, which include controlling the mud flow and cuttings’ removal, changing shaker screens regularly, and cleaning the screens with pressure washers. Occasionally, the drill floor workers also visit the mud pit and the mud pump areas (OGP/IPIECA, Drilling Fluid Task Force 2009; Kirkhus et al. 2015).

Referents were selected from work groups without any known regular exposure to occupationally generated particulate matter or other airborne chemical contaminants. They were employed as managers, administrative personnel, technicians, radio operators, medics, catering personnel (cooks excluded), deck crews, mechanics and production workers. All the examined workers were of Caucasian ethnicity, mostly Scandinavians.

Part 2 of the study was designed as a cross-sectional study of drill floor workers, who were examined with HRCT of the lungs. Inclusion was restricted to males employed as drill floor workers in the time window between 1980 and 1990 and who had been previously exposed to mud during offshore oil drilling for at least 3 years. Lists of all the drill floor workers employed between 1980 and 1990 were provided by the three offshore companies engaged in oil drilling in the Norwegian offshore sector that were approached and agreed to participate. The lists included 338 names supplemented with the date of birth and last known residential address. Of these, 20 individuals were either deceased, living abroad or the current address was unknown. Altogether, 118 subjects with residential address within acceptable (< 100 km) travel distance to the two radiology institutions in the Norwegian cities of Stavanger and Bergen were identified.

Recruitment of participants started with the workers employed in 1980. Potential participants were first informed by letter and subsequently recruited by telephone. The goal of examining at least 50 participants was reached, when 92 workers had been contacted by telephone. Of these, 57 participants were included, while 35 persons could not or did not wish to participate. Ten persons informed by mail were not reached by telephone. Sixteen eligible subjects were not informed by mail, because the target of recruiting 50 participants had been reached. The study was approved by the South East Regional Ethical Committee for Medical Research, Norway (REK 2009/186 and REK 2011/1605d). Participation in the study was voluntary. An informed written consent was obtained from all the participants.

### Pulmonary function

Pulmonary function of the currently exposed drill floor workers included in Part 1 of the study was measured with a spirometer (PowerCube^®^—Diffusion, Ganshorn Medizin Electronic GmbH, Niederlauer, Germany), according to the American Thoracic Society (ATS)/European Respiratory Society (ERS) guidelines (Miller et al. 2005). The following variables were recorded: forced vital capacity (FVC) and forced expiratory volume in 1 s (FEV_1_). The ratio FEV_1_/FVC was calculated. The same two experienced technicians performed all the spirometric measurements. Two experienced physicians assessed all the measurements, and the best out of at least three tests was used in the statistical calculations. The spirometer was calibrated daily. The height and weight of the participants were measured.

### High-resolution computer tomography

In Part 2 of the study, low-dose thin-section CT images of the lungs without intravenous contrast material were obtained following identical protocol in scanning the participants in both the institutions. Scans were obtained in the supine position during breath-holding. Section thickness was 1–1.2 mm, while scan intervals were 10 mm for inspiratory and 20 mm for expiratory images. A high-spatial-frequency (bone) algorithm was used for reconstruction of images. Tube current settings were adjusted according to body weight, but with low-dose references. The HRCT images were initially reviewed by radiologists at the two institutions to reveal any pathology with immediate need for medical attention. The electronic files with the images were then transferred to the Department of Radiology and Nuclear Medicine at Oslo University Hospital. The images were reviewed in random order by two experienced chest radiologists (TMA and AG, with 21 and 12 years of experience, respectively) on a picture archiving and communication system (PACS) screen in consensus without any information collected from the participants by the questionnaire. The presence and extent of airways disease and interstitial lung disease, including ground-glass opacity, airspace consolidation, reticular pattern, bronchiectasis, nodules and air trapping, were evaluated. The criteria for evaluation of the severity of HRCT abnormalities have previously been reported (Hansell et al. 2008; Aaløkken et al. 2012). Sub-segmental air trapping comprising less than 5% of the lung parenchyma was considered normal (Tanaka et al. 2003).

### Questionnaires

Background data were collected by a self-administered validated questionnaire in Part 1 of the study (Kongerud et al. 1989). The questions included the occurrence of respiratory symptoms, smoking and smokeless tobacco (snuff) habits and information on the current and previous diseases. The participants were categorized as current, former or never smokers. In addition, time since and type of the last nicotine intake before the health examinations were recorded.

A self-constructed questionnaire assessing the occupational history and exposures, smoking habits, and known diseases or medication was used in Part 2 of the study.

### Nicotine and cotinine in serum

In order to control for smoking status and and for potential differences in smoking habits offshore and at home, nicotine and cotinine in serum were measured among the participants in Part 1 of the study. Blood samples were collected from the cubital vein with 10 mL BD Vacutainers without additives (Becton, Dickinson and Company, Franklin Lakes, NJ, USA). Serum was separated by centrifugation at 2000*g* for 15 min and pipetted into 4.0 mL NUNC^®^ polypropylene cryotubes (Thermo Fisher Scientific, Watham, MA, USA). The samples were kept frozen at − 20 °C, until its long-term storage at − 80 °C at the National Institute of Occupational Health. Sample preparation and measurements have been described in detail (Ellingsen et al. 2006). However, in this study, a different internal standard (cotinine-(methyl-d_3_)) was used to improve the within- and between-assay precisions of the analytical method. The detection limits (DL) were 7.3 ng nicotine/mL and 1.7 ng cotinine/mL. The DL was defined as 3 × standard deviation of the blanks.

### Occupational exposure

#### Air sampling and measurements

Details on air sampling and measurements among currently exposed drill floor workers participating in Part 1 of the study have been published (Kirkhus et al. 2015). Air samples were collected by personal sampling in the breathing zone of the drill floor workers and outside personal protective respirators, if used. No air samples were collected among referents.

Oil mist was collected on glass fiber filters in 37 mm closed-face cassettes (CFC) (EMD Millipore Corporation, Billerica, MA, USA), and oil vapor was collected by charcoal tubes connected to the CFCs at an air flow rate of 1.4 L/min. Non-volatile mud components (NVM) were collected on polyvinyl chloride filters (PVC502500, EMD Millipore Corporation) in 37 mm Millipore cassettes, and elemental carbon (EC) and organic carbon (OC) were collected in 37 mm standard aerosol cassettes fitted with pre-heated quartz filters (Pallflex Tissue Quartz 2500 QAT-UP, Pall Corporation, Port Washington, NY, USA). The NVM and OC/EC sampling cassettes were fitted with thoracic cyclones (CK 2.69, BGI Instruments, Waltham, MA, USA) and operated at an air flow rate of 1.6 L/min.

Explosion proof air pumps were used (SKC 224-PCMTX4 and SKC 224-PCTX4, SKC Inc. Eighty-Four, PA, USA). Before and after each sampling period air flow rates were measured with a calibrated rotameter (Brooks 2–65 MM, Emerson Electric Co., Hatfield, PA, USA). Cassettes and tubes were sealed and stored in refrigerators immediately after sampling. The EC and OC samples were sent to Sunset Laboratory Inc. (Tigard, OR. USA) for analysis. The other samples were analyzed at NIOH.

Information on the number of days of drilling could be obtained from four oilrigs with 29 drill floor workers included in Part 1 of the study, while no exposure measurements were collected in Part 2.

### Statistics

Group differences at baseline were analyzed with Student’s *t* test for continuous variables and with the Chi square test for categorical data. Student’s *t* test was also used to analyze baseline differences between participants who were examined at follow-up and those who did not attend that examination. A univariate general linear model was used to adjust for differences in age, height and weight between groups.

Cross shift changes in pulmonary function were analyzed by linear mixed models including both fixed and random effects (lme function in R, nlme package, version 3.1–122). The inclusion of a random intercept for a worker allowed us to take into account the dependency of repeated observations. As fixed effects, in addition to the group variable (referent or exposed), also age, height and weight were included together with the concentrations of nicotine and cotinine in serum.

**Fig. 1:**
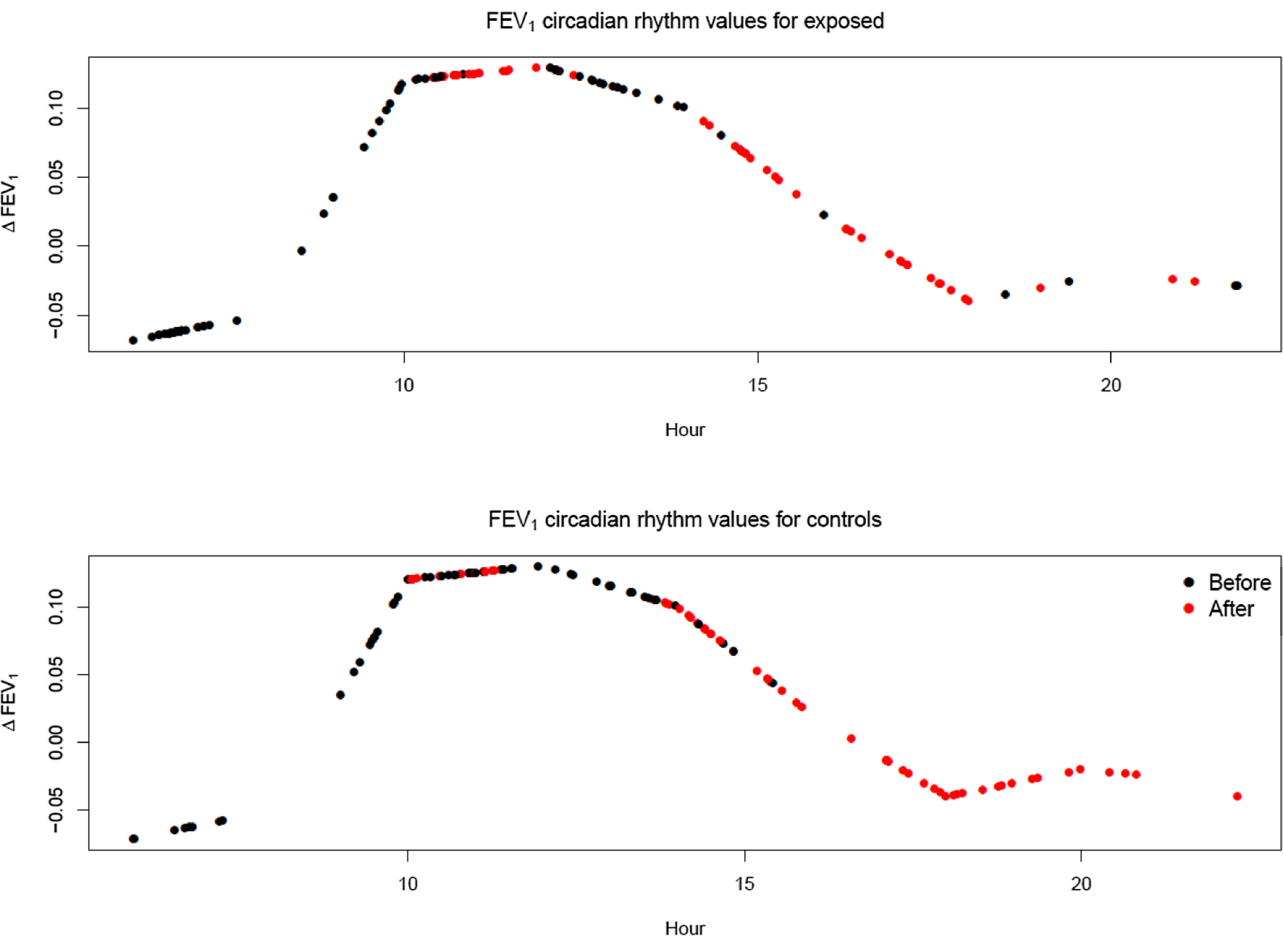
Estimated deviations from the 24 h mean for FEV_1_ due to circadian rhythm (Spengler and Shea 2000). The dots show the circadian rhythm deviations for all the FEV_1_ measurements. A given dot shows the assumed circadian rhythm deviation for a particular observation at its time of measurement

To assess the possible impact of circadian rhythm on pulmonary function, FVC and FEV_1_ were adjusted for circadian rhythms using estimates from one study particularly designed to assess circadian alterations in pulmonary function (Spengler and Shea 2000). The diurnal variations of FEV_1_ and FVC in that study do not follow a simple cosinus/sinus curve, or a mathematical curve easily described in few parameters. Thus, due to the lack of original data, Fig. 1 from that study was magnified and the distance from baseline to each plot was carefully measured. The adjusted variables (FVC and FEV_1_) were re-analyzed using the same mixed model analysis described above. The circadian rhythm adjustments were given as delta values, representing deviations from the 24 h mean, for every 2 h and between those time points, linear interpolation was used. Adjustment for circadian rhythm was then made by subtracting FVC and FEV_1_ by these interpolated delta values. Figure 1 illustrates the effect of circadian rhythm on FEV_1_. A value of 0.10 around 10 a.m. means that, due to circadian rhythm, the FEV_1_ value at that particular time will be expected to be 0.10 higher than the 24 h—average FEV_1_ value.

All the mixed model analyses were done in *R*, version 3.2.2. The statistical package SPSS (version 21) was used for all the other statistical analyses. A two-tailed *p* value < 0.05 was considered to be of statistical significance.

## Results

### Part 1

The currently exposed drill floor workers were significantly younger than the referents, and the prevalence of current smokers was higher. At baseline, pulmonary function and prevalence of the self-reported symptom “wheezing in the chest” were comparable between the two groups (Table 1). Air samples for oil mist, oil vapor and airborne mud measurements were collected by personal sampling and showed median concentrations of 0.18, 14 and 0.14 mg/m^3^, respectively (not tabulated). However, drilling was carried out for, on average, 7.3 days of the 14-day work period among those 29 drill floor workers, where such information was available.

**Table 1:** Characteristics and pulmonary function at baseline among the 65 currently exposed drill floor workers and 65 referents included in Part 1 of the study

	Drill floor workers (*n* = 65) Mean (range)	Referents (*n* = 65) Mean (range)
Age (years)^a^	30 (19–59)	46 (30–69)
Height (cm)	180.7 (169–196)	181.5 (169–196)
BMI (kg/m^2^)	26.1 (20.7–39.3)	26.6 (20.2–36.8)
Years offshore ^a^	5.8 (0.5–32)	14 (0–35)
Current smokers (%)	31	23
Former smokers (%)	21	26
Never smokers (%)	48	51
S-nicotine in smokers (µg/L)	14.0 (< DL-53)	20.4 (< DL-54.2)
S-cotinine in smokers (µg/L)	205 (< DL-382)	236 (4–434)
Self-reported asthma (%)	3	3
Morning cough (%)	9	6
Wheezing in the chest (%)	23	29
FVC^c^ (L)^b^	4.9 (3.3–7.3)	5.0 (2.9–6.3)
FEV_1_^d^ (L)^b^	3.9 (2.8–5.4)	3.9 (2.4–5.1)
Ratio FEV_1_/FVC	0.80 (61–94)	0.78 (51–90)

^a^ *p* < 0.05

^b^Adjusted for age, height and weight

^c^Forced vital capacity

^d^Forced expiratory volume in 1 s

Twenty-four subjects were lost to follow-up, of whom 14 were exposed and 10 were referents. No statistically significant differences in lung function, age, height, BMI, serum nicotine or cotinine at baseline between those who were lost to follow-up and the remaining participants were observed.

**Fig. 2:**
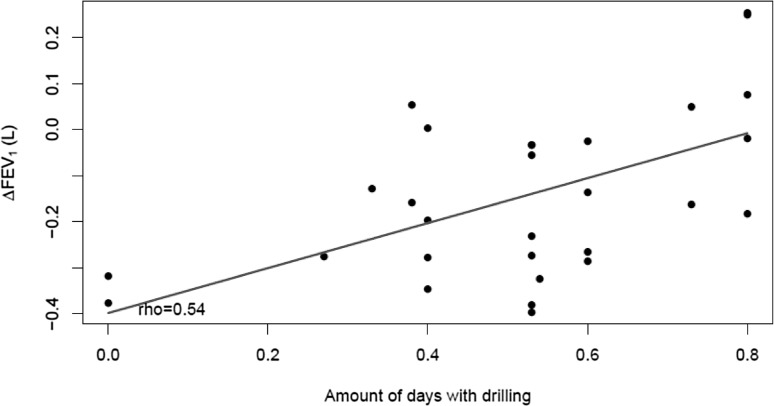
The association between the difference in FEV_1_ between baseline and follow-up adjusted for diurnal variation (ΔFEV_1_) among the 29 currently exposed offshore drill floor workers participating in Part 1 of the study and the time with active drilling expressed as the ratio between number of days with active drilling and the 14-day work period (amount of days with drilling). Spearmans’s rho = 0.54, *p* = 0.002

Statistically significant declines in FEV_1_ and FVC were observed during the 14-day work period in both the groups (Table 2). The declines were similar in smokers and non-smokers (not tabulated). However, the drill floor workers were examined significantly earlier than the referents at baseline, the mean examination times being 10:25 a.m. and 11:08 a.m. (*p* = 0.001), respectively. Drill floor workers were also examined earlier at follow-up, the respective mean times being 14:17 p.m. and 15:48 p.m. (*p* = 0.08). After adjusting for these differences in examination times, no statistically significant declines in FEV_1_ and FVC were found among the referents across the 14-day work period. However, the statistically significant decline in FEV_1_ remained among the drill floor workers. The difference in the decline of FEV_1_ decline between drill floor workers and referents did not quite attain statistical significance (*p* = 0.086). Differences in smoking habits onshore and offshore, as assessed by serum nicotine and cotinine, had no significant impact on FVC and FEV_1_ across the 14-day work period. The decline in FEV_1_ was associated with number of days of drilling activity among drill floor workers with available information (Spearman’s rho = 0.54; *p* = 0.002) (Fig. 2), suggesting larger decline in FEV_1,_ when less drilling was carried out.

**Table 2:** Differences in pulmonary function (in L) between baseline and follow-up after a 14-day work period offshore among currently exposed drill floor workers and referents participating in Part 1 of the study, adjusted for age, height, weight, nicotine and cotinine

		Drill floor workers (*n* = 65/51)^a^				Referents (*n* = 65/55)^a^				Drill floor workers vs referents			
	Outcome	Estimate	Lower	Upper	*p*	Estimate	Lower	Upper	*p*	Estimate	Lower	Upper	*p*
Panel A	FEV_1_	− 0.06	− 0.11	− 0.02	0.0096	− 0.07	− 0.11	− 0.02	0.0034	0.01	− 0.06	0.07	0.85
	FVC	− 0.05	− 0.10	0.00	0.043	− 0.06	− 0.11	− 0.02	0.0099	0.01	− 0.06	0.08	0.74
Panel B	FEV_1_	− 0.09	− 0.14	− 0.03	0.002	− 0.02	− 0.07	0.03	0.46	− 0.07	− 0.15	0.01	0.086
	FVC	− 0.04	− 0.10	0.01	0.15	− 0.01	− 0.06	0.04	0.67	− 0.03	− 0.11	0.05	0.44

Panel A: not corrected for circadian rhythm; Panel B: corrected for circadian rhythm

^a^Number of observations before and after the offshore work period with complete covariate information

### Part 2

Background data of the participants in Part 2 of the study are presented in Table 3. The median time of exposure to mud as a drill floor worker was 5.4 years. In addition, 42 of the subjects had also been exposed to mud in other jobs in the drill crew for a median of 6 years. Pulmonary HRCT examinations showed abnormalities in 54% of the participants (Table 4). One subject presented with subpleural reticular pattern without cysts and superimposed ground glass opacities representing fine intralobular fibrosis with overall extent of 5–10%. Coarse fibrosis with microcystic pattern or honeycombing was not observed. Moderate centrilobular emphysema (less than 5% overall extent) was detected in 11% of the subjects. All were current or former smokers [mean smoking years 32 (range 7–47 years)]. Nineteen subjects (33%) had findings indicative of small airways disease. However, air trapping comprising more than 25% of the lung parenchyma was not observed. None of the eight subjects with air trapping reported lung disease, but five were current or former smokers.

**Table 3:** Background data on 57 former drill floor workers examined with high-resolution computer tomography of the lungs in Part 2 of the study

	%	Median	Range
Years as drill floor workers		5.4	1.0–28
Years in other mud exposed position (*n* = 42^a^)		6.0	1.0–31
Pooled mud exposed years		11	3.4–34
Age at HRCT examination		54	43–65
Current smokers	26		
Former smokers	42		
Never smokers	32		
Years of smoking among ever smokers		26	2.5–50
Self-reported diseases			
Obstructive pulmonary diseases	9		
Earlier pneumonias	4		
Pulmonary injuries	2		
Rheumatoid diseases	5		
Cancers	5		

Data missing for two participants

^a^Derrick man (*N* = 32), mud processing operator (*N* = 3), pump man (*N* = 3), not specified (*N* = 4)

**Table 4:** Results from high-resolution computer tomography of the lungs of 57 former offshore drill floor workers examined in Part 2 of the study

	N (%)^a^
Normal HRCT	26 (46)
Lung fibrosis: fine intralobular without evident cysts	1 (2)
Lung fibrosis: microcystic reticular pattern	0 (0)
Lung fibrosis: macrocystic reticular pattern with honeycombing	0 (0)
Emphysema	6 (11)
Small airways disease (air trapping, bronchiectasis)	19 (33)
Nodules	1 (2)
Other incidental benign findings (centrilobular micronodules, perifissural micronodules, small atelectasis, lung cysts, pleural thickening, pleural fluid)	20 (35)

^a^Some subjects had more than one finding. *N* (percent)

## Discussion

### Part 1

This is to our knowledge the first scientific study to address pulmonary health among offshore drill floor workers. Lung function measurements at baseline were carried out at an earlier time of the day than at follow-up, due to the time schedule of the helicopter transport to and from the oil rigs. After correction for the examination time difference, FEV_1_ declined statistically significantly across the 14-day work period among the drill floor workers, but not among the referents. Contrary to the á priori hypothesis, the decline was most pronounced, when there was less active drilling and thereby less mud circulation.

Diurnal variation in pulmonary function is well-known (Guberan et al. 1969; Hetzel and Clark 1980; Borsboom et al. 1999; Spengler and Shea 2000). In particular, the study by Spengler and Shea (2000) that was specifically designed to assess diurnal variation, showed a substantial increase in FEV_1_ from 8:00 a.m. to 10:00 a.m. (approximately 200 mL) and a gradual decline from 14:00 p.m. to 18:00 p.m. Also, Borsboom et al. (1999) reported substantial improvement in FEV_1_ from 9.00 a.m. to around noon, followed by a gradual decrease at 8:00 p.m. Based on their estimation of FEV_1_ in a subject aged 44.5 years, the difference in FEV_1_ among the persons examined at 9 a.m. or 11 a.m. would be around 70 mL. The procedure of helicopters transporting crews offshore before returning the old crews onshore may have resulted in a systematic difference between baseline and follow-up in the time of lung function measurements and thus affected the amount of decline in pulmonary function between baseline and follow-up.

There are no standard methods for correction of diurnal pulmonary variation. As shown by Spengler and Shea (2000), the diurnal variations in FEV_1_ and FVC do not follow a simple cosinus/sinus curve, or a mathematical curve easily described in few parameters. To estimate the diurnal variations of our data, we could have used an additive mixed model approach, where the diurnal variation is described by a smooth flexible curve. However, we had too few observations and too much variability in the data to possibly estimate such a flexible curve with precision. Therefore, we used the corrections for circadian rhythms given by Spengler and Shea (2000). Because of the substantial improvement in FEV1 in the morning, it is important to consider diurnal variation in epidemiological studies. They estimated the peak to trough of mean circadian change of FEV_1_ to 3.5%, while Borsboom et al. (1999) estimated a mean circadian change in FEV_1_ of 2.8% (86 mL). These estimates are well within the mean decline in FEV_1_ of 60 and 70 mL among the drill floor workers and referents, respectively, before correction for diurnal variation. After correction, the drill floor workers had a statistically significant mean FEV_1_ decline of 90 mL across the 14-day work period, in contrast to a non-significant mean decline of 20 mL among the referents. The difference in FEV_1_ decline between drill floor workers and referents did not reach statistical significance (*p* = 0.086), but this may be due to the limited sample size. However, a slight occupational exposure cannot be excluded among the referents. Such exposure may have reduced the differences in pulmonary function between the two groups. Regression analysis suggested that the decline in FEV_1_ was substantially smaller, when active drilling was performed than when no active drilling took place (Fig. 2). This could indicate that decreased pulmonary function among the drill floor workers was not associated with exposure to oil mist from circulating mud. This suggestion is further substantiated by their low exposure of oil mist and oil vapor (median concentrations of 0.18 and 14 mg/m^3^, respectively), when compared to national occupational exposure limits of 0.6 and 30 mg/m^3^ (12 h TWA). Moreover, the available data suggest that active drilling occurred for around 7 days on average. Confidential drilling data were not available from all the companies, which is a limitation of the study.

The drill floor workers were also exposed to mud components other than oil (median 0.14 mg/m^3^). Some mud components are known irritants to the skin and eyes, and both oil-based and water-based muds are alkaline solutions (Hansen et al. 1991; Saeed et al. 1997). The process of drilling consists of different phases, and one of the involved drilling companies has estimated that the mud pumps run for approximately 25% of the time during drilling operations (personal information). In periods without circulation of mud, the drill floor workers are usually not working in the shaker area. Work tasks then typically involves running in or pulling out the drill stem, maintenance and assembling of drilling equipment. Pulling out the mud-filled drill stems leads to variable degrees of mud spills at the drill floor, depending on equipment design. Cleaning up spills of mud, oil and grease are frequent work tasks for drill floor workers during non-drilling periods. The contaminated surfaces are usually sprayed with an alkaline detergent, before the solution is removed by pressure washing. As shown in shaker rooms, pressure washing may lead to aerosol generation from the substances that are removed by the cleaning process (Kirkhus et al. 2015). The cleaning agents used are described as irritating to the airways. It is imaginable that such exposures may impact pulmonary function during non-drilling periods. Potential chemical exposures during non-drilling periods were not assessed, but should be addressed in future studies. Health examinations were carried out at the heliport at Sola airport that is a hub for transport of personnel to and from the offshore sector. Many participants live far away from this hub, and many of them travel by plane between their home and the heliport. Often, the time between helicopter transport and commuting flights is limited. We assume that this has contributed to the loss to follow-up of 26 subjects. These subjects did not differ from the other participants with respect to important background variables. Thus, this loss to follow-up has most likely not systematically distorted the results, but resulted in a lower study power than originally planned. However, the use of linear mixed models should partly account for this loss of power, as incomplete observations can also be included in such analyses.

Offshore workers have their health examined every second year to assess their fitness for work. This could suggest that subjects with a disease may stop working offshore. This potential selection of particularly healthy workers is not likely to bias the results of Part 1 of the study, as pulmonary function decline across a 14-day work period was the outcome. Moreover, the potential dilution of the reference group with former drill floor workers is small, because the highly specialized knowledge of the drill team workers are less required in other departments of the rig. The exposed workers have long working hours, but potential fatigue should be evenly distributed in both the groups. There were also no signs of the impact of fatigue in the study of Spengler and Shea (2000). It may also be possible that helicopter transportation for up to 2 h prior to the second examination may have had some impact on pulmonary function, but this would eventually affect the results equally in the two groups.

### Part 2

Examinations with HRCT showed minor signs of pulmonary fibrosis in one subject only. The former drill floor workers had been exposed during a period with substantially higher exposure to oil mist, than what had been the case for the participants in Part 1 of the study. An arithmetic mean concentration of 4.3 mg/m^3^ measured by personal sampling was reported for the period 1989–1997 (Steinsvåg et al. 2006).

It has been suggested that exposure to oil mist may cause pulmonary fibrosis in cable workers exposed to oil mist levels of 0.5–1.0 mg/m^3^, and in animals exposed to cable oils (Skyberg et al. 1986, 1990, 1992), but only minor signs of pulmonary fibrosis were detected among the 57 examined subjects. One subject with subpleural reticular pattern without cysts and superimposed ground glass opacities representing fine intralobular fibrosis with an overall extent of 5–10% had additional pleural plaques and small rounded atelectasis and parenchymal bands, indicative of previous asbestos exposure and asbestosis. This does not exclude the possibility that fibrosis may occur after oil mist exposure, but at such high exposure levels, it is at least a rare event.

Signs of small airways disease were present in 33% of the drill floor workers participating in Part 2 of the study. A study of air trapping on HRCT images in asymptomatic subjects showed a prevalence of 52%, while the prevalence for subjects aged 51–60 years was 65% (Lee et al. 2000). Thus, air trapping is common, and cannot be attributed to oil mist exposure among the drill floor workers. It should, however, be noted that only employees who had worked in the three larger companies participated in Part 2 of the study. It is possible that the exposure levels of the drill floors in these companies may have been low compared to the other companies, which could result in an underestimation of a potential development of pulmonary fibrosis. However, we do not have any such information. Also, of the 118 eligible drill floor workers, only 57 participated. Disease status, including pulmonary fibrosis, is unknown for the non-participating subjects.

In conclusion, after correction for diurnal variation in pulmonary function, the drill floor workers experienced a decline in FEV_1_ across the 14-day work period offshore. There were, however, no indications of an association with oil mist exposure. The possible impact of exposure of the other airborne contaminants should be further explored. No indications of interstitial lung diseases related to oil mist exposure in drill floor workers were observed.
